# Care-associated adverse events related to the use of laser in urological interventions: the French experience

**DOI:** 10.3389/fruro.2025.1507018

**Published:** 2025-03-28

**Authors:** Maher Abdessater, Frédéric Panthier, Philippe Michel, Vanessa Avrillon, Bertrand Pogu, Stéphane Bart

**Affiliations:** ^1^ Risk Management Department, French Association of Urology (AFU), Paris, France; ^2^ Urology Department, NOVO Hospital, Pontoise, France; ^3^ Urology Department, Tenon Hospital-Sorbonne University, Assistance Publique – Hôpitaux de Paris (APHP), Paris, France; ^4^ Urology Department, Hospital of Chalons en Champagne, Chalons en Champagne, France

**Keywords:** care-associated adverse events, laser, urology, operating room safety, surgical safety

## Abstract

**Introduction:**

The use of laser in urology is increasing, especially in renal stones and benign prostatic hypertrophy. Despite the interest in this technology on improving surgical management, several adverse events may result. This work collates French reports of care-associated adverse events (CAEs) resulting from lasers used in urological interventions.

**Materials and methods:**

This is a collection of CAEs between May 2016 and December 2023 declared by urologists involved in accreditation throughout France. These CAEs were classified according to five levels of severity. They have been described and classified according to the ALARM protocol. The statistics were mainly descriptive. Fisher’s exact test and Student’s *t*-test were used via the software R.

**Results:**

Between May 2016 and December 2023, between the 1,376 declared events, 149 laser-related CAEs were reported in urological interventions. Five CAEs (3.4%) were classified as grade 3, and six CAEs (4%) were classified as grade 4. All the other CAEs were between grades 1 and 2 with negligible consequences. The immediate reported causes of laser AEs were the clinical complexity of the case (7.38%), the technical gesture (14.1%), patient information (24.83%), material (38.25%), and medications (15.43%). Incidents caused by problems in material seem to be more frequent in younger patients (*p* < 0.001), healthier patients (ASA 1) (*p* = 0.003), risky situations (*p* < 0.001), and laser procedures (*p* < 0.001).

**Conclusion:**

In France, 7.4% of CAEs related to the use of laser in urological surgery are of major to critical severity. Training teams on the use of laser generators and providing feedback on functional results and related specific morbidity seem necessary to guarantee the proper use of these technologies and the safety of staff and patients.

## Introduction

In all surgical specialties, the use of laser can provide many benefits for the patients, such as decreased postoperative pain, decreased postoperative surgical site infections, improved wound healing, precise cutting, and blood loss reduction (1).

Surgeons and physicians use different types of lasers, including neodymium-doped yttrium aluminum garnet (Nd : YAG), erbium:YAG, holmium:YAG, carbon dioxide (CO_2_), diode, and argon in a variety of specialties (e.g., urology, oncology, cardiology, neurology, ophthalmology, and dermatology) (2).

As laser technology continues to evolve, the number of procedures for which lasers may be used is also increasing, offering surgeons new ways to perform their interventions with safer patient outcomes (2).

On the other hand, despite all these benefits, risk factors of laser use are not negligible and can sometimes be redoubtable. Fire, physiologic damage to the eyes and other tissues, and other biological hazards such as laser plume were described in the literature and addressed by safety standards (3).

In the field of surgery, urologists were among the first motivated specialists to adopt these new technologies into their clinical and surgical practice, and the first reported use of the laser technique in urology dates back to 1976 with Staethler et al. who used a flexible quartz fiber light guide to assess the depth of tissue removal without the risk of perforation of the bladder wall (4).

Since then, laser techniques have progressively gained a major place in the endoscopic treatment for patients with benign prostatic hyperplasia, bladder or upper urinary tract tumors, urolithiasis, urinary tract strictures, and also for lesions of the external genitalia (5–8).

Thus, laser devices became powerful tools with an expanding use in urology; however, they have the power to permanently harm patients. This is why laser surgeries should be performed by trained medical professionals in an adequate medical setting, providing higher quality care with a lower risk of adverse effects and litigation (9).

Urologists and other healthcare providers should be aware of the risks of complications of laser surgery. Although early recognition and treatment of complications can help decrease the sequela of side effects, prevention is still the mainstay of these surgeries. Therefore, the use of lasers needs to be constantly monitored to improve the safety of patients and healthcare workers (9).

The aim of the present article is to collate French reports of adverse events resulting from lasers used in urological interventions, in order to find and propose solutions for patients’ and healthcare workers’ safety.

## Materials and methods

The French High Authority for Health (HAS) implements a system for accrediting doctors and medical teams. This accreditation is a voluntary risk management process based on the declaration of adverse events associated with care in order to improve the quality of care and the safety of patients.

The accreditation program includes actions that assess and improve practices and evaluate risk management by collecting and analyzing care-associated adverse events (CAEs). The analysis of these anonymously declared events leads to the production of individual and/or collective recommendations by the HAS. We used the HAS database to collate all CAEs during urological procedures, including those using lasers between May 2016 and December 2023.

All the collated CAEs have been categorized and graded according to their severity. In our protocol, the Common Terminology Criteria of Adverse Events (CTCAE) scale was utilized. In CTCAE, an adverse event is defined as any abnormal clinical finding temporally associated with the use of a therapy; causality is not required.

We used the version 5.0 (v5.0) of the scale that was updated in 2018 (10). The severity of CAEs was graded as follows:

Grade 1: mild; asymptomatic or mild symptoms; clinical or diagnostic observations only; no intervention indicated.
Grade 2: moderate; minimal, local, or non-invasive intervention indicated; limiting age-appropriate instrumental activities of daily living (ADL).
Grade 3: severe or medically significant but not immediately life-threatening; hospitalization or prolongation of hospitalization indicated; disabling; limiting self-care ADL.
Grade 4: life-threatening consequences; urgent intervention indicated.
Grade 5: death related to CAE.

The elements linked to these CAEs have been described and classified by their causes and circumstances according to the clinical risk unit and association of litigation and risk management (ALARM) protocol (11).

To identify factors that may have contributed to the adverse events declared in the “laser procedures,” we compared them to those declared in “non-laser” procedures. CAEs with laser use (group 1) were compared to those with no laser use (group 2).

In order to have more homogeneous groups, we only kept the interventions where the laser device is sometimes used. Therefore, similar procedures were compared, with the difference of using (or not) the laser.

The protocol received approval from the institutional review board, and informed consent from the participants was waived because this is a non-interventional study based on regular healthcare data.

### Statistical analysis

The statistics were mostly descriptive. Discrete data were described by their frequency expressed as a percentage with their 95% confidence interval and were compared by Fisher’s exact test. Confidence intervals were only obtained after angular transformation. Numerical data were described by their mean (with their 95% confidence interval calculated by bootstrap) and standard deviation. Continuous data were compared by Student’s *t*-test after checking for equality of variances. The statistics were produced using the R software (R Core Team 2021) (12).

## Results

Between May 2016 and December 2023, 1,376 CAEs were reported in urological interventions through the HAS database. Of these, 149 were declared in procedures using laser devices.

The laser-related AEs were in the following interventions: 30 in laser enucleations of the prostate (holmium and thulium) (20.1%), 93 in flexible and rigid ureteroscopies with laser management of the stones (62.4%), and 26 in GreenLight laser vaporizations of the prostate (17.5%).

The main causes of these CAEs in laser procedures were failure to manage anticoagulants (*N* = 15/10.1%), inappropriate antibiotic prophylaxis (*N* = 5/3.4%), communication failure in perioperative situations (*N* = 23/15.4%), 1-day surgery incidents (*N* = 23/15.4%), unavailable preoperative lab tests (*N* = 5/3,4%), defective or unavailable equipment or material (*N* = 71/47.7%), inappropriate therapeutic strategy (*N* = 3/2.05%), and lack of healthcare professionals’ competency (*N* = 4/2.7%).

Five CAEs (3.4%) were classified as grade 3, and six CAEs (4%) were classified as grade 4. Concerning grade 3 CAEs, we found a case of per- and postoperative bleeding after holmium laser enucleation of the prostate (HoLEP) for benign prostatic hyperplasia (BPH) in an 87-year-old patient requiring blood transfusion, a case of sepsis after HoLEP for BPH in a 73-year-old patient that necessitated transfer to the ICU, a case of sepsis after ureteroscopy for renal stones in a 70-year-old patient that also required transfer to the ICU, a case of pubic symphysitis after photoselective vaporization of the prostate (PVP) GreenLight laser for BPH in a 59-year-old patient managed with long-duration IV antibiotics, and a case of postoperative bleeding after PVP GreenLight laser for BPH in a 72-year-old patient that required blood transfusion.

Concerning grade 4 CAEs, we noted a case of cerebrovascular accident (CVA) after ureteroscopy for renal stones in an 82-year-old patient that required thrombolysis; a case of postoperative bleeding after ThuLEP for BPH in an 85-year-old patient managed with endoscopic coagulation in the operating room; a case of postoperative bleeding after HoLEP for BPH in a 79-year-old patient, also treated with endoscopic coagulation in the operating room; a case of burn of the distal urethra after HoLEP for BPH in a 59-year-old patient that required proximal urethrostomy; a case of urethro-rectal fistula after PVP GreenLight laser for BPH in a 65-year-old patient that required York–Mason intervention; and a case of urethro-pubic fistula after PVP GreenLight laser for BPH in a 58-year-old patient that required Bricker ileal conduit diversion.

All the other CAEs were classified between grades 1 and 2 with negligible consequences. The details of the severity of the CAEs are provided in **Table 1**. Concerning demographic data, no difference was found between group 1 and group 2 (**Table 2**).

**Table 1:** Severe and life-threatening CAEs related to laser interventions in HAS database.

Age (years)	Disease	Initial Intervention	Complication	CTCAE scale	Urgent action/ reintervention
87	BPH	HOLEP	Per and post-operative Bleeding	3	Blood transfusion
82	Renal stone	Ureteroscopy	CVA	4	Thrombolysis
73	BPH	HOLEP	Sepsis	3	Transfer to ICU
85	BPH	ThuLEP	post-operative Bleeding	4	Endoscopic Coagulation in the O.R
79	BPH	HOLEP	post-operative Bleeding	4	Endoscopic Coagulation in the O.R
70	Renal stone	Ureteroscopy	Sepsis	3	Transfer to ICU
59	BPH	HOLEP	Burn of the distal urethra	4	Proximal Urethrostomy
65	BPH	PVP greenlight laser	Urethro-rectal fistula	4	YORK MASON intervention
59	BPH	PVP greenlight laser	Pubic symphysitis	3	Long duration IVAB
58	BPH	PVP greenlight laser	Urethro-pubic fistula	4	Bricker ileal conduit diversion
72	BPH	PVP greenlight laser	Post-operative bleeding	3	Blood transfusion

BPH, Benign Prostatic Hypertrophy; HOLEP, Holmium Laser Enecluation of the Prostate; CVA, Cerebro-Vascular Accident; ICU, Intensive Care Unit; ThuLEP, Thulium Laser Enucleation of the Prostate; PVP, Photo-Vaporization of the Prostate; IVAB, intravenous antibiotics; O.R, operating room.

**Table 2:** Demographic data.

	Laser interventions (group 1)	Other interventions (group 2)	p
Sex - Female - Male	30/149 (20.13%) 119/149 (79.87%)	93/298 (31.2%) 205/298 (68.8%)	0.055
Age (Years)	64.7 (SD=13.9)	63.8 (SD=14.8)	0.956
BMI - Underweight - Normal weight - Overweight - Obese	0/149 (0%) 55/149 (36.91%) 66/149 (44.29%) 28/149 (18.8%)	10/298 (3.35%) 112/298 (37.58%) 133/298 (44.63%) 43/298 (14.44%)	0.0721
Pregnancy	5/149 (3.35%)	16/298 (5.36%)	0.566
ASA Score - 1 - 2 - 3	67/149 (44.97%) 50/149 (33.56%) 32/149 (21.47%)	150/298 (50.34%) 92/298 (30.87 %) 56/298 (18.79%)	0.737
Surgical complexity - Easy case - Normal case - Difficult case	79/149 (53.02%) 48/149 (32.21%) 22/149 (14.77%)	221/298 (74.16%) 58/298 (19.46%) 19/298 (6.38%)	0.08

The laser AEs’ immediate reported causes related to the intervention itself were classified according to the ALARM protocol as follows: the clinical complexity of the case (7.38%), the technical gesture (14.1%), patient information (24.83%), material (38.25%), and medications (15.43%). A significant difference (*p* = 0.016) between the two groups was only found in the material category (**Table 3**).

**Table 3:** AEs causes related to the intervention itself according to the ALARM protocol.

	Other interventions (control group)	Laser interventions (group 1)	p value
Clinical complexity	32/298 (10.73%)	11/149 (7.38%)	0.912
Technical gesture	45/298 (15.12%)	21/149 (14.1%)	0.08
Patient information	103/298 (34.56%)	37/149 (24.83%)	1.13
Material and sterilization	62/298 (20.8%)	57/149 (38.25%)	**0.016**
Medications	56/298 (18.79%)	23/149 (15.43%)	0.82

Bold values means statistically significant.

We compared the causes linked to material and sterilization to other causes of CAEs (**Table 4**): The incidents caused by problems in material seem to be more frequent in younger patients (*p* < 0.001), healthier patients (ASA 1) (*p* = 0.003), risky situations (*p* < 0.001), and laser procedures (*p* < 0.001).

**Table 4:** Comparison between the causes related to material and sterilization and other causes of CAEs.

	Material and sterilization	Other causes	P value
**Age (years)**	59.4 (+/- 14.8)	66.6 (+/-14)	**p < 0.001**
**BMI** - Underweight - Normal weight - Overweight - Obese	0/149 (0%) 39/149 (24.16%) 70/149 (46.98%) 40/149 (26.86%)	3/298 (1.01%) 115/298 (38.59%) 135/298 (45.30%) 45/298 (15.10%)	p = 0.763
**ASA Score** - 1 - 2 - 3	102/149 (68.45%) 39/149 (26.18%) 8/149 (5.37%)	183/298 (61.41%) 64/298 (21.47%) 51/298 (17.12%)	**p = 0.003**
**Surgical complexity** - Non complex - Complex - Very complex (Hard)	129/149 (86.57%) 19/149 (12.76%) 1/149 (0.67%)	251/298 (84.23%) 45/298 (15.10%) 2/298 (0.67%)	p = 0.921
**Aim of the intervention** - Diagnostic - Therapeutic	1/149 (0.67%) 148/149 (99.33%)	11/298 (36.92%) 287/298 (73.08%)	p = 0.37
**Level of emergency** - Programmed intervention - Urgent intervention	123/149 (82.55%) 26/149 (17.45%)	257/298 (86.24%) 41/298 (13.76%)	**p < 0.001**
**Laser use** - Yes - no	80/149 (53.69%) 69/149 (46.31%)	229/298 (76.85%) 69/298 (23.15%)	**p < 0.001**

BMI, Body mass index; ASA, American Society of Anesthesiologists.

Bold values means statistically significant.

## Discussion

Lasers are routinely used in endourologic procedures; therefore, AEs associated with their use are often declared. In this study, we collated the laser-associated AEs using the French HAS database to assess their safety in urological surgery.

Fortunately, most of the reported CAEs (92.6%) were mild to moderate with no considerable consequences on the patients. However, 3.4% of the CAEs were severe or medically significant but not immediately life-threatening and led to prolonged hospitalization. In addition, 4% of them were life-threatening and resulted in urgent intervention.

Among the grade 3 CAEs, bleeding and sepsis were the most redoubtable complications. Bleeding was mostly associated with BPH endoscopic procedures and sepsis was associated with ureteroscopies. According to De Corninck et al., the worst complication of ureteroscopy is urosepsis (13). The use of antibiotic prophylaxis, treatment of preoperative UTI, and low procedural time seem to reduce the risk of this complication (14).

Among the grade 4 CAEs, we reported a symphyseal fistula after GreenLight photovaporization of the prostate; this complication led to a surgical treatment with Bricker conduit urinary diversion after conservative treatment failure. The same complication has been reported by Garrido-Abad et al. who tried, without success, to treat a patient with transurethral catheter placement for 3 months and 6 weeks of antibiotic therapy (15).

All these findings underline the urgent need for dramatic changes in the organizational culture of the healthcare system and surgeons‘ practices in order to prevent death and injury from errors related to laser use in the operating room.

The percentage of CAEs related to HoLEP and prostate vaporization was almost similar in our database; this is in line with the findings of El Shal et al. who reported similar perioperative complications and the need for auxiliary procedures in these groups (16).

Most of the reported AEs (62.4%) were related to ureteroscopies. In fact, it has been shown that when lasers are activated with a deflected fiber in a tight bending radius, fiber failure can occur with consequent irradiation of the ureteroscope resulting in instrument damage and possibly patient injury (17). Thus, failure to confine energy emission to the tip of the laser can result in damage to equipment and injury to patients and operating room personnel. Therefore, it is not surprising that most of the reported AEs were related to ureteroscopies where the smallest and most fragile fibers are used.

The material problems were the only significant cause of CAEs in our study (38%, *p* < 0.001); these problems can result from generator failure or laser fiber and scope damage. Optical fiber breakage or tip detachment put the patient at risk of burns or infection if they become lodged in tissue. Optical fibers, though flexible, have not been designed to bend to acute degrees during procedural manipulations and are vulnerable to damage (18). Thus, urologists have to be careful when using optical fibers and should be sufficiently competent in using the laser equipment after suitable training.

Furthermore, taking into consideration the significant variability of the commercially available laser fibers, thermal breakdown is much lower with the non-tapered larger diameter laser fibers compared with the tapered 200-lm core fibers used with Ho : YAG lasers (19). Another study found that for Ho : YAG laser, sub-300-lm fibers were associated with significantly higher connector end failures with the laser generator compared with 365-lm fibers (4% vs. 0%; *p* < 0.001) (20).

Concerning the adequate use of fibers, before using the optical fiber, it should be checked that it is not damaged and is firmly attached to the laser output aperture. Before firing, the fiber’s distal end has to be put in its intended position. Once in position, it may be appropriate to secure the fiber by fixing it to the endoscope to prevent it from moving during use. Inadequate cleaning of the optical filter during the procedure may cause it to overheat (21). This information and others have to be integrated into the training of every urologist who performs laser interventions.

Besides the interpretation of these reported CAEs, we would insist that accurate and reliable data on adverse events are essential for protecting public health, as it helps regulatory agencies, healthcare providers, and manufacturers identify, assess, and mitigate risks. To achieve this level of accuracy, mandatory reporting systems have proven crucial by ensuring that all relevant cases of adverse events are captured in a structured, timely, and comprehensive manner. Voluntary reporting systems, which rely on healthcare providers or patients to submit reports when they choose to, often lead to underreporting due to lack of motivation, awareness, or time. This underreporting creates gaps in adverse event data, potentially delaying important safety updates or product recalls. Mandatory systems, on the other hand, enforce reporting through legal requirements, making it harder for events to be overlooked.

Mandatory reporting ensures a more systematic and uniform approach to data collection, reducing inconsistencies and biases. When reporting is a requirement rather than an option, data collected tend to reflect a more accurate depiction of risks, allowing for more reliable analysis. Mandatory systems enable health authorities to track patterns or clusters of adverse events, making it easier to detect emerging risks. This can lead to earlier interventions such as label changes, dosage adjustments, or product withdrawals, reducing harm to patients.

Mandating reporting increases accountability among healthcare providers and medical institutions by ensuring that they take their role in patient safety seriously. This can foster a culture of safety where healthcare workers feel responsible for identifying and reporting potential risks.

Several countries have implemented effective mandatory reporting systems, demonstrating their impact on improving the accuracy and reliability of adverse event data: The U.S. Food and Drug Administration (FDA) operates several mandatory reporting mechanisms. Australia’s Therapeutic Goods Administration (TGA) mandates that healthcare professionals and manufacturers report adverse events related to therapeutic goods, including medical devices, pharmaceuticals, and vaccines. The country’s “Adverse Event Monitoring System” has been instrumental in ensuring quick responses to safety concerns, as seen during their proactive approach to identifying side effects of COVID-19 vaccines.

Norway mandates reporting through its centralized pharmacovigilance system, which integrates adverse event reporting across medical professionals, patients, and manufacturers. Japan has a stringent mandatory reporting system requiring pharmaceutical companies and healthcare professionals to report adverse drug reactions and events related to medical devices.

### Limitations

This study relies on voluntary reporting of CAEs; this fact can introduce underreporting bias, as healthcare professionals may not report all CAEs due to time constraints, lack of awareness, or other factors. This finding emphasizes the need for initiatives that encourage a culture of mandatory and comprehensive reporting.

The lack of granular data on laser types, operative techniques, and surgeon experience restricts our ability to draw more specific conclusions. Future studies with the inclusion of specific laser-related variables in accreditation systems would better facilitate data analysis.

## Conclusion

Despite their great advantages, laser interventions can be very dangerous. Fortunately, the most reported adverse events are related to material damage, but patient harm (although less frequent) remains the main concern.

These findings highlight the need to support laser safety education and training for all healthcare personnel working with or around laser systems. In France, one of the main pillars of this safety program is the team accreditation and certification provided by the HAS.

Future studies that collect more variables to better understand their influence on CAEs should be done, including studies that compare the safety and efficacy of laser procedures with alternative treatment options to provide a more comprehensive risk–benefit assessment.

## Data Availability

The original contributions presented in the study are included in the article/supplementary material. Further inquiries can be directed to the corresponding author.
